# Correction: Bottom-up and top-down influences at untrained conditions determine perceptual learning specificity and transfer

**DOI:** 10.7554/eLife.20672

**Published:** 2016-08-18

**Authors:** Ying-Zi Xiong, Jun-Yun Zhang, Cong Yu

Xiong Y-Z, Zhang J-Y, Yu C. 2016. Bottom-up and top-down influences at untrained conditions determine perceptual learning specificity and transfer. *eLife* **5**:e14614. doi: 10.7554/eLife.14614.

Published July 05, 2016

In the published article the image and legend for Figure 4 was incorrectly labelled as Figure 5 and vice versa.

The article has been corrected accordingly.

